# An expeditious and facile method of amyloid beta (1–42) purification

**DOI:** 10.1371/journal.pone.0307213

**Published:** 2024-07-11

**Authors:** Md. Aminul Haque, Il Seon Park

**Affiliations:** 1 Department of Biomedical Sciences, Chosun University, Dong-gu, Gwangju, Korea; 2 School of Pharmacy, BRAC University, Merul Badda, Dhaka, Bangladesh; 3 Research Lab, Rufaida BioMeds, Aftabnagar, Dhaka, Bangladesh; 4 Department of Cellular and Molecular Medicine, Chosun University, Dong-gu, Gwangju, Korea; Nathan S Kline Institute, UNITED STATES OF AMERICA

## Abstract

For the study of amyloid beta (Aβ) associated toxicity which is supposed to be the main pathological agent in Alzheimer’s disease (AD), it is important to secure Aβ peptide with appropriate biological activity. However, commercial and synthetic Aβ often have some pitfalls like less cell toxicity, prompt aggregation and excess price, using recombinant technology, these issues can be resolved though the method also suffered from some problems such as low yield, aggregation and prolong time to purify. Thus, we previously developed an easy, economic and convenient method for Aβ42 purification using highly expressed GroES-Ubiquitin-Aβ42 fusion protein. The method was efficient, but further development was performed to improve the procedure and increase the yield. Focus was on the isolation of the fusion protein (GroES-Ubiquitin) from Aβ42 peptide. After a series of systematic testing with several chemicals, we found that methanol could precipitate efficiently the fusion protein, while the Aβ peptide was recovered in the supernatant. By this method, Aβ peptide was easily purified without tedious chromatographic steps which are main obstacles to purify the peptide in the previous method. This method yielded ~20 mg highly pure Aβ42 peptide from 1-liter bacterial culture. Different biophysical characterizations and bioactivity assays indicate that the peptide purified using this method was competitive with others which have been previously reported whereas considering the simplicity, final yield and time of purification, this method is the optimal solution.

## Introduction

Accumulating evidence suggests that the amyloid-β peptide (Aβ) plays a key role in the pathogenesis of Alzheimer’s disease [1]. 36–43 amino acids containing amyloid-β (Aβ) peptide is formed upon sequential proteolysis of the amyloid precursor protein [2, 3]. Among different available Aβ sequences, Aβ40 is the most common one [4] but Aβ42 peptide which has two more amino acids Ile and Ala to the C-terminus of Aβ40 is responsible for diseases [5]. Aβ peptides’ structure naturally transforms into unstable β-sheet-rich intermediate structures which interact to form oligomers, protofibrils, and fibrils which play role in disease progression and cell to cell transmissibility [6, 7].

A clear exploration of the structure of different aggregation of Aβ may help to find out proper treatment guideline to suppress the formation of toxic Aβ aggregations. Though after exploration of the sequence of Aβ, much progress has been made but due to the lack of a reliable source of Aβ peptide, structural analysis of Aβ monomer and its aggregates has been retarded. Usually, Aβ peptide is synthesized chemically [8] but this process is time-consuming and expensive [9, 10]. Moreover, chemical synthesis of aggregation susceptible peptides is still persisting as a critical issue [11]. On resin agglomeration leads to decreased yield, chemical and physical heterogeneity (typical reason for lack of reproducibility) is a significant issue to stay away from chemical synthesis of peptide, which may lead scientists to a confused state [12]. Many of these difficulties may have been avoided by using fusion protein consisting of fusion partner, a cleavage site and expected peptide [13]. Purification of recombinant Aβ has been studied previously [14, 15] but almost in every case either highly specialized equipment or expensive reagents are required [12, 14]. Some methods are only applicable for biologically insignificant Aβ species [16].

To facilitate Aβ peptide associated research, here we explain a very easy, fast, economical and highly efficient procedure for the purification of recombinant Aβ42 peptide. In this study highly expressed GroES-Ubiquitin-Aβ42 fusion protein has been used and the responsible vector is constructed in our lab earlier [15]. Most of the recombinant Aβ peptide purification techniques include chromatographic [14, 15, 17, 18] or ultrafiltration technique [19] for the isolation purposes. This method is quite new and interesting for the purification of recombinant Aβ42 peptide without using any chromatographic or filtration technique. Importantly, freeze drying step is also eliminated in this method.

## Materials and methods

### Materials

Fetal Bovine serum (FBS) was obtained from Atlas Biologicals Inc. (Fort Collins, USA). Dulbecco’s modified Eagles medium (DMEM) and Penicillin/Streptomycin (P/S) were purchased from Welgene (Gyeongsangbuk-do, Korea). Urea and Phosphate buffer saline (PBS) were bought from Georgia Chem. & Equip. Co. Inc. (Norcross, USA), and Ameresco (Framingham, MA, USA), respectively. Methanol and Ethanol were from OCI Company Ltd. (Seoul, Korea) and Isopropyl β-D-1-thiogalactopyranoside (IPTG) was from Bioneer (Daejon, Korea). All other chemicals were procured from Sigma-Aldrich (St. Louis, MO, USA), unless otherwise mentioned.

### Expression of GroES-Ubiquitin-Aβ42 fusion protein

Previously constructed pET28b-GroES-ubiquitin-Aβ42 vector was used and the fusion protein was expressed as described earlier [15]. Briefly, protein expression was induced with 0.4 mM IPTG when OD600 reaches at ~0.6, and was additionally cultured for 4 h. After harvesting, cells were lysed in a lysis buffer (20 mM Tris–Cl, pH 8.0, 150 mM NaCl, 0.1 mM PMSF, 0.1 mM EDTA and 1 mM β-mercaptoethanol) and centrifuged for 1 h at 11000 x g. Due to the overexpression of GroES-ubiquitin-Aβ42 fusion protein, inclusion body was formed. Inclusion body was solubilized in a solubilization buffer (50 mM Tris–Cl, pH 8.0, 150 mM NaCl, 1 mM DTT and 6 M urea). Centrifugation at 36000 x g for 30 min was carried out to remove insoluble proteins. Supernatant was saved for further purification process.

### Purification and preparation of Aβ42 peptide

Saved supernatant was two-fold diluted with a buffer (50 mM Tris–Cl, pH 8.0, 150 mM NaCl, and 1 mM DTT) to maintain urea concentration of 3M to avoid precipitation of fusion protein and incubated with Usp2-cc enzyme at 1:100 enzymes to substrate molar ratio at 37˚C for 3 h. After digestion, 100% methanol was added at 1:1 ratio to sample and kept for 10 min at room temperature. Sample was centrifuged for 10 min at 2000 x g at 4˚C. Supernatant was collected and dried by rotary evaporator at 30˚C. The dried fraction was washed with 100% ethanol to remove the salts and centrifuged again at 2000 x g for 10 min. This step was repeated three times, followed by a final wash with 100% methanol and centrifugation at 2000 x g for 10 minutes. The pellet containing pure peptide was collected and dissolved in 1,1,1,3,3,3-hexafluoro-2-propanol (HFIP). Dissolved peptide was centrifuged at 2000 x g for 10 min at 4˚C and supernatant was saved and aliquoted. HFIP was evaporated and peptide was stored at -20˚C for long term preservation. Prior to use, monomerized Aβ42 peptide was dissolved in 0.1% NH_4_OH at a concentration of 2 mg/ml and sonicated for 10 min. The solution was diluted to the desired concentration with PBS or cell culture media. Aβ42 oligomer was prepared as previously described [20, 21]. Purity of the purified peptide was checked using RP-HPLC (Shimadzu Corporation, Kyoto, Japan) as previously described [14]. Briefly, 150 μl peptide solution (2 mg/ml) was injected into a C_18_ column (4.6 mm × 250 mm × 5 μM) (Grace Vydac, Hesperia, CA, USA) where buffer A (10 mM ammonium acetate, pH 10 in 2% acetonitrile) and buffer B (70% acetonitrile) were used at a flow rate of 1 ml/min using the following linear gradient of buffer B (0%-20% over 5 minutes, 20%-40% for next 30 minutes and 40%-100% for next 25 minutes). The wavelength of the detector was set at 220 nm. Mass of the purified peptide was further examined at a commercial peptide company (Anygen Co., Seongnam, Korea) where Shimadzu Biotech Axima Assurance was used where linear mode and 2.67 mV were applied.

Usp2-cc enzyme was prepared based on previous study upon slight modification [22]. Briefly, *E*. *coli* BL21 pLysS transformed with previously constructed pET15b-Usp2-cc vector was used to express Usp2-cc enzyme in LB media in presence of ampicillin antibiotic. Protein expression was initiated with 0.4 mM IPTG when the OD600 reached approximately 0.6, and then the culture was continued for an additional 4 h at 30˚C. For harvesting 1 liter culture, 20 ml STE buffer (100 mM NaCl, 10 mM Tris–Cl, pH 8.0, 1 mM EDTA) was used and the pellets were lysed using 15 ml lysis buffer (20 mM Tris–Cl, pH 8.0, 10 mM NaCl, 0.1 mM PMSF, 0.1 mM EDTA and 1 mM DTT). Lysed sample was centrifuged at 11000 x g at 4˚C for 1 h and supernatant was collected and filtered using a 0.45μ syringe filter before proceeding with purification using Ni^2+^-NTA column chromatography. Column was equilibrated with a buffer (20 mM Tris–Cl, pH 8.0, 300 mM NaCl, 1 mM β-mercaptoethanol, and 10% glycerol), sample was loaded into the column and washed with the same buffer. Finally, elution buffer (20 mM Tris–Cl, pH 8.0, 300 mM NaCl, 1 mM β-mercaptoethanol, 10% glycerol, and 250 mM imidazole) was used to elute the Usp2-cc enzyme and stored at -80˚C. ~150 mg Usp2-cc enzyme was obtained from 6-liter culture over the course of 2 days which is sufficient enough to digest GroEs-Ubiquitin-Aβ42 fusion protein from ~60 liters cultures.

### Biophysical characterization of purified Aβ42 peptide

Fibrillogenesis, secondary structure analysis and protofibril-fibril formation were studied using ThT-fluorescence assay, circular dichorism (CD) spectroscopy and transmission electron microscopy (TEM), respectively, as previously described [2].

Briefly, for fibrillogenesis, incubated (37°C) 20 μL of 20 μM peptide solution were properly mixed with freshly prepared 5 μM thioflavin T (ThT, Bioneer, Daejeon, Korea) at each time point and the subsequent fluorescence was measured on a microplate spectrofluorometer Gemini-XS (Molecular Devices, San Jose, CA, USA) at an excitation wavelength of 445 nm and emission wavelength of 490 nm. Both the peptide and ThT solutions were prepared in phosphate buffer saline (PBS).

For CD spectroscopy, 20 μM peptide solutions were prepared in PBS and the spectra were measured just after incubating at 37°C using Jasco J-810 Spectropolarimeter (Jasco Co., Gunma, Japan) at 25°C where cuvette with 1 mm path length at 0.5 nm intervals between 190 and 250 nm was used. At 0.1 nm resolution, 0.5 s response time, and 50 nm/min scan speed, five cumulative readings were obtained.

Freshly prepared Aβ42 peptide (2 mg/mL) in NH_4_OH was used as previously described [21] to form oligomer and fibril. The peptide solution was properly mixed into serum free DMEM (without phenol red) and kept at 4°C for 24 hours at 100 μM concentration for oligomer formation. After incubation, peptide solution was centrifuged for 15 minutes at 16000 × g and supernatant were isolated and diluted to 20 μM with serum free DMEM. For fibril preparation, 20 μM peptide solution in PBS was arranged and kept at 37°C for 24 hours. For TEM, 5 μL peptide solution of 20 μM was taken on a Formvar-coated 200-mesh nickel grid (SPI Supplies, West Chester, PA, USA) and after 5 minutes, upon three times washing with distilled water, samples were stained with 2% uranyl acetate. The TEM (H-7600, Hitachi, Tokyo, Japan) was used to analyze the grids and it was run at an accelerating voltage of 80 kV and a magnification of 40,000×.

### Bioactivity investigations of purified Aβ42 peptide

The human epithelial HeLa cells (obtained from Korean Cell line Bank) were used for different bioactivity assays. MTT and alamarBlue assays were used to check the cell viability. 10μM Ac-DEVD-amino-methyl-coumarin (AMC) (AG Scientific, Inc., San Diego, CA, USA) was used to measure the DEVDase activity and the interaction of Aβ42 and caspase-9 was assessed using Carl Zeiss LSM510 microscope (Germany). All the experiments were done as previously described [2].

Concisely, for the cell viability assays, cells (15 × 10^3^) were seeded, cultured, serum deprived and treated according to the desired plans at 37°C. 20 μL 3-(4,5-dimethylthiazol-2-yl)-2,5-diphenyltetarzolium bromide (MTT) solution and 100 μL solubilization buffer [20% sodium dodecyl sulfate (SDS) solution in 50% (*v*/*v*) N, N-dimethylformamide (DMF) (pH 4.7)] were used for the MTT assay. With the use of microplate reader (KisanBio, Seoul, Korea), absorbance was measured at 570 nm. For alamarBlue assay, 10 μL of alamarBlue (Life Technologies, Inc., Carlsbad, CA, USA) was used and fluorescence was recorded at excitation and emission wavelengths of 560 and 590 nm, respectively, using a Gemini-XS microplate spectrofluorometer (Molecular Devices, San Jose, CA, USA).

For caspase assay, cells (2 × 10^4^) were seeded, incubated, serum-starved and treated with samples at 37°C. Then, ice-cold PBS was used to rinse the cells twice. Subsequently, 40 μL of lysis buffer (20 mM HEPES-NaOH, pH 7.0, 1 mM EDTA, 1 mM EGTA, 20 mM NaCl, 0.25% Triton X-100, 1 mM DTT, 1 mM PMSF, 10 μg/mL leupeptin, 5 μg/mL pepstatin A, 2 μg/mL aprotinin, and 25 μg/mL N-acetyl-Leu-Leu-Norleucinal) was added to each well and kept on ice for 20 min. Caspase assay buffer (20 mM HEPES-NaOH, pH 7.0, 20 mM NaCl, 1.5 mM MgCl2, 1 mM EDTA, 1 mM EGTA and 10 mM DTT) and Ac-DEVD-amino-methyl-coumarin (AMC) (AG Scientific, Inc., San Diego, CA, USA) were then added to the mixture. The level of AMC was noted for 1 h at 5 min intervals at excitation and emission wavelengths of 360 and 480 nm, respectively, using a Gemini-XS microplate spectrofluorometer (Molecular Devices, San Jose, CA, USA).

For confocal microscopy, cells (1 × 10^5^) were taken, cultured with and without serum and treated with the Aβ42 peptides. Upon setting the treated cells in methanol at -20°C, cells were permeabilized with 0.3% Triton X-100. Mouse monoclonal anti-A antibody 6E10 (BioLegend, San Diego, CA, USA) or rabbit polyclonal anti-caspase-9 (p10) antibody (Santa Cruz Biotechnology, Santa Cruz, CA, USA) were added to each sample and incubated overnight at 4°C after being blocked overnight with 0.1% bovine serum albumin. Alexa-Fluor-546-TRITC-conjugated goat anti-rabbit IgG and Alexa-Fluor-488-FITC-conjugated goat anti-mouse IgG antibodies (dilution, 1:200, Invitrogen, Waltham, MA, USA) were introduced and kept for 2 hours at room temperature after washing with PBS. DAPI in Vectashield mounting medium (Vector Laboratories, Burlingame, CA, USA) was applied to stain the nuclei. Confocal images were taken with a Carl Zeiss LSM510 microscope (Germany) using the manufacturer’s software (LSM 510). For each confocal channel with a resolution of 2048 2048 pixels, four different variable pinholes (97 M) of 1.0 airy units were employed. A plan apochromat 63 × 1.4 oil immersion objective was used to focus the cells.

## Results

### Expression and purification of Aβ42 peptide

**Fig 1:**
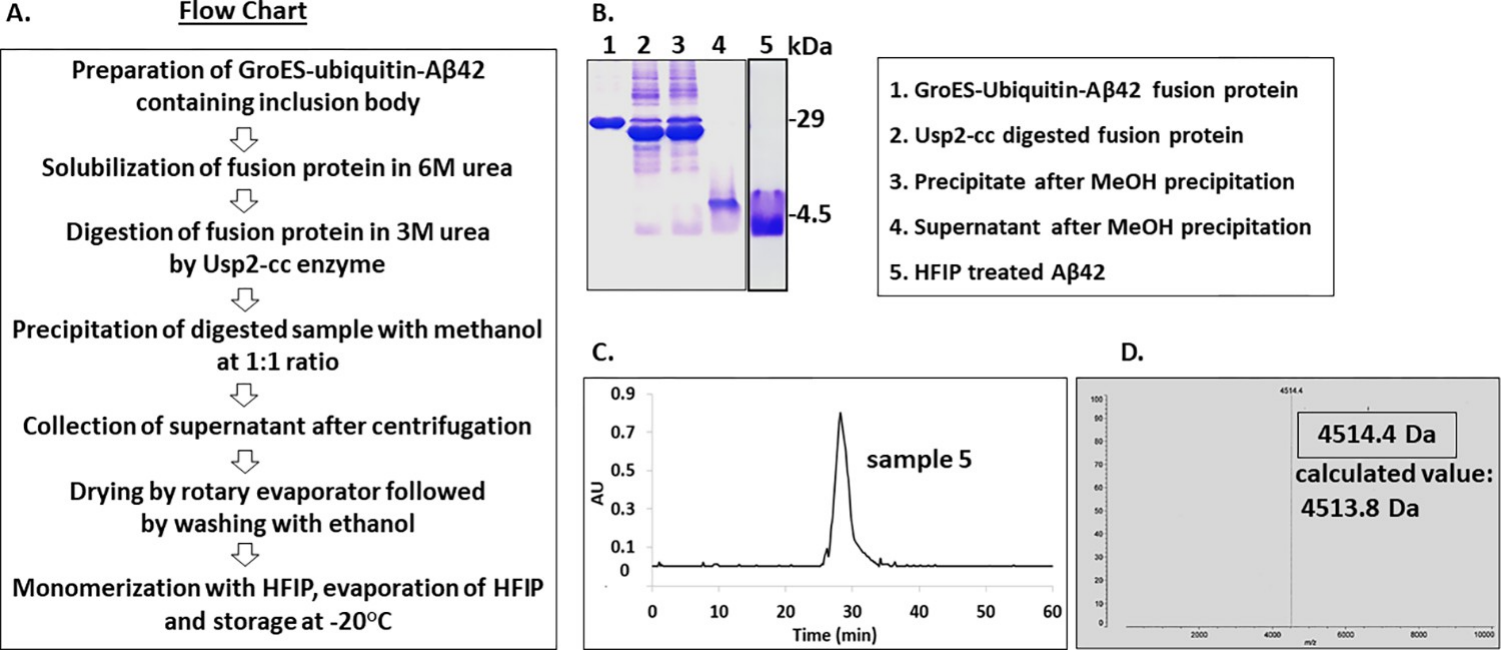
Purification and purity analysis of Aβ42. (A) Schematic representation of purification method. (B) SDS-PAGE gel analysis of different fractions; Lane 1, GroEs-ubiquitin-Aβ42 fusion protein; Lane 2, Usp2-cc digested fusion protein; Lane 3 and 4, precipitate and supernatant fractions after methanol precipitation, respectively; Lane 5, HFIP treated Aβ42. (C) Purity checking of purified peptide by RP-HPLC using C18 column. (D) Confirmation of correct molecular mass of purified Aβ42 peptide by mass spectroscopy.

600 mg GroES-ubiquitin-Aβ42 fusion protein was isolated from 1-liter bacterial culture as inclusion body. The inclusion body was dissolved initially in 6 M urea containing buffer and later the digestion reaction was set with Usp2-cc enzyme at 1:100 enzyme to substrate molar ratio for 3 h at 37°C where urea concentration was 3 M. Cleavage of Aβ42 peptide was examined using 12% SDS-PAGE. Previously, several chromatographic procedures like affinity column chromatography, SEC, HPLC or molecular weight cut off membrane filters were applied to isolate the cleaved Aβ42 peptide. Here, based on the solubility profile of Aβ42 peptide and fused GroES-ubiquitin protein, Aβ42 was purified from the mixture. Different surfactants, several pH, huge number of buffers, different combinations of aqueous and organic solvents were used to find out an appropriate one where cleaved Aβ42 and GroES-ubiquitin will show different solubility pattern. Finally, it was found that in presence of 50% methanol and 1.5 M urea, the Aβ42 peptide remains at dissolved state whereas the fused GroES-ubiquitin does not. So, after digestion, methanol was added to the digested sample at 1:1 ratio to make the final methanol concentration 50% and urea concentration 1.5 M. Way of methanol addition is a crucial step here. To get the better separation and better yield, methanol was added slowly with continuous stirring. Sudden addition of methanol without stirring will lead to the precipitation of Aβ42 also which is not expected. Now, the challenge was removal of methanol, urea and other salts present in the system. Usually for this purpose, chromatographic techniques or molecular weight cut off membrane filters or desalting methods are used which are expensive, time consuming and troublesome. Here, in this method, solvent was evaporated using rotary evaporator at 37°C where RPM was 100 and after drying, in the dried sample, peptide along with urea and other salts were present. Different aqueous and organic solvents including water, methanol, ethanol, propanol, butanol, acetonitrile etc. were used to dissolve the urea and other salts. Except ethanol, others were not useful to serve our purpose. In case of water and methanol, along with urea and other salts, Aβ42 peptide was also got dissolved and using propanol, butanol, acetonitrile and other organic solvents, the removal of salt and urea was not good enough. Finally, washing with the 100% ethanol (where purity of ethanol was 98%) facilitated the complete removal of urea and salts but that did not allow the Aβ42 peptide to come into the solution. Dried samples were washed with ethanol three times and then washed again using methanol. After washing, the sample was centrifuged to get the pellets of highly pure Aβ42 peptide and finally it was dissolved in HFIP, aliquoted and evaporated for the long-term preservation. From 1-liter bacterial culture, approximately 20 mg pure Aβ42 peptide was recovered. The purification procedure is summarized as a flowchart (Fig 1A). Purity of fusion protein, digested protein and purified Aβ42 peptide were analyzed several times during the whole purification procedure using SDS-PAGE (Fig 1B and S1 Raw images), RP-HPLC was used to confirm and compare the purity (Fig 1C and S2 Fig) with HPLC purified Aβ42 peptide. Mass of the purified peptides were also analyzed (Fig 1D and S1 Fig). RP-HPLC and MS data of our peptides were found comparable with HPLC purified peptide (S1 and S2 Figs).

### Biophysical characterization of purified Aβ42 peptide

**Fig 2:**
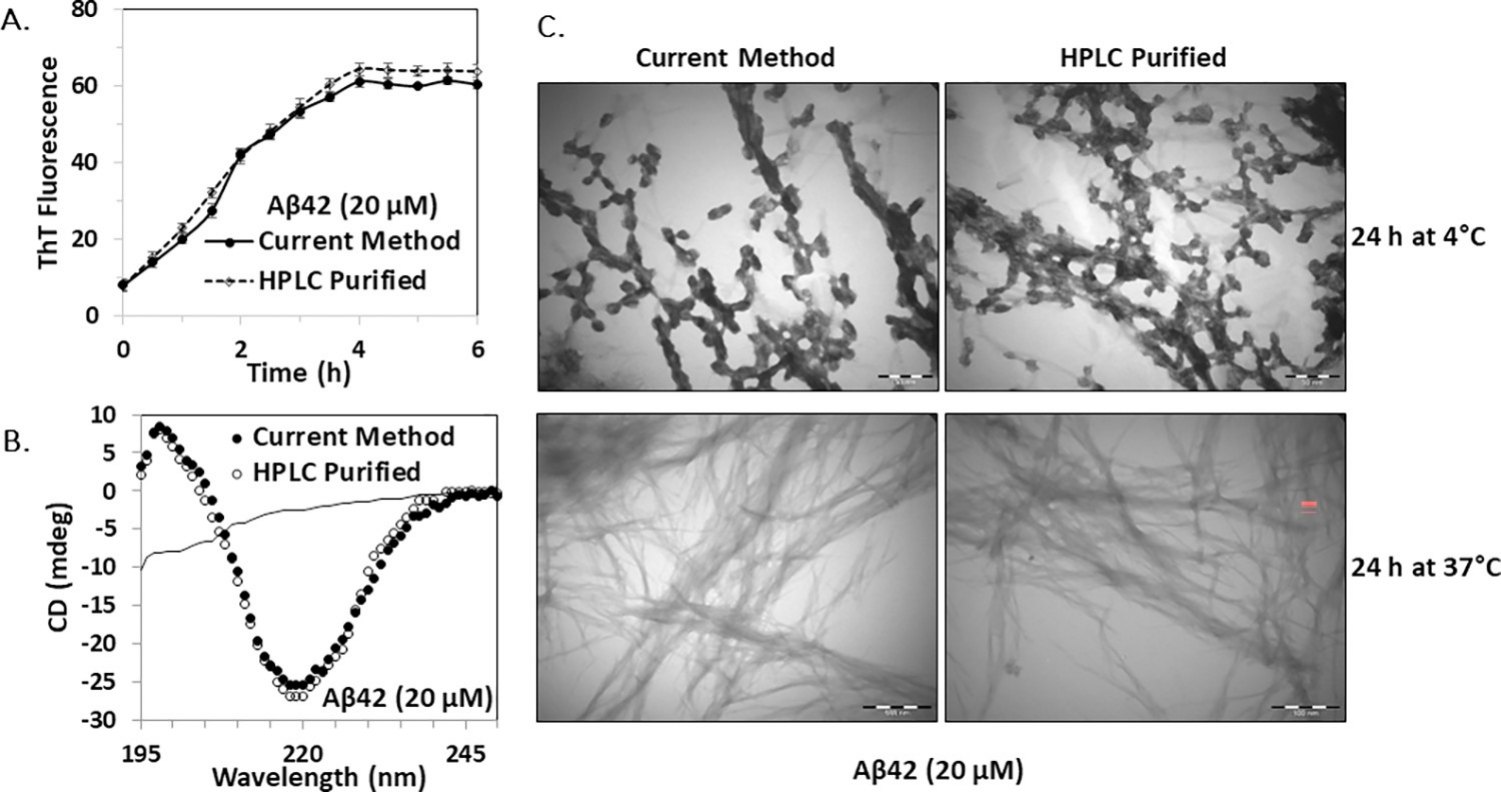
Biophysical characterization of purified Aβ42 peptide. (A) Fibrillogenesis by time-dependent ThT-fluorescence assay using 20 μM Aβ42 peptides. RFU represents relative fluorescence unit. (B) CD spectra analysis of 20 μM freshly prepared Aβ42 sample (solid line) and aggregated Aβ42 samples where filled and empty circles represent peptide purified by current method and HPLC method, respectively. (C) Protofibril and fibril formation were assessed by collecting images with TEM at 40000×. Scale bars represent 100 nm in TEM images. Triplicate experiments were performed and standard deviations were indicated as bars.

Different biophysical properties of purified Aβ42 peptide were analyzed and compared with the HPLC purified form. Fibrillogenesis kinetics was assessed by ThT assay (Fig 2A), β-sheet formation was analyzed by CD spectroscopy (Fig 2B) and protofibril and fibril formation were examined by TEM (Fig 2C). Results of these properties were in line with the HPLC-purified peptides and previous reports [14, 18, 23, 24].

### Assay of different biological properties of purified Aβ42 peptide

**Fig 3:**
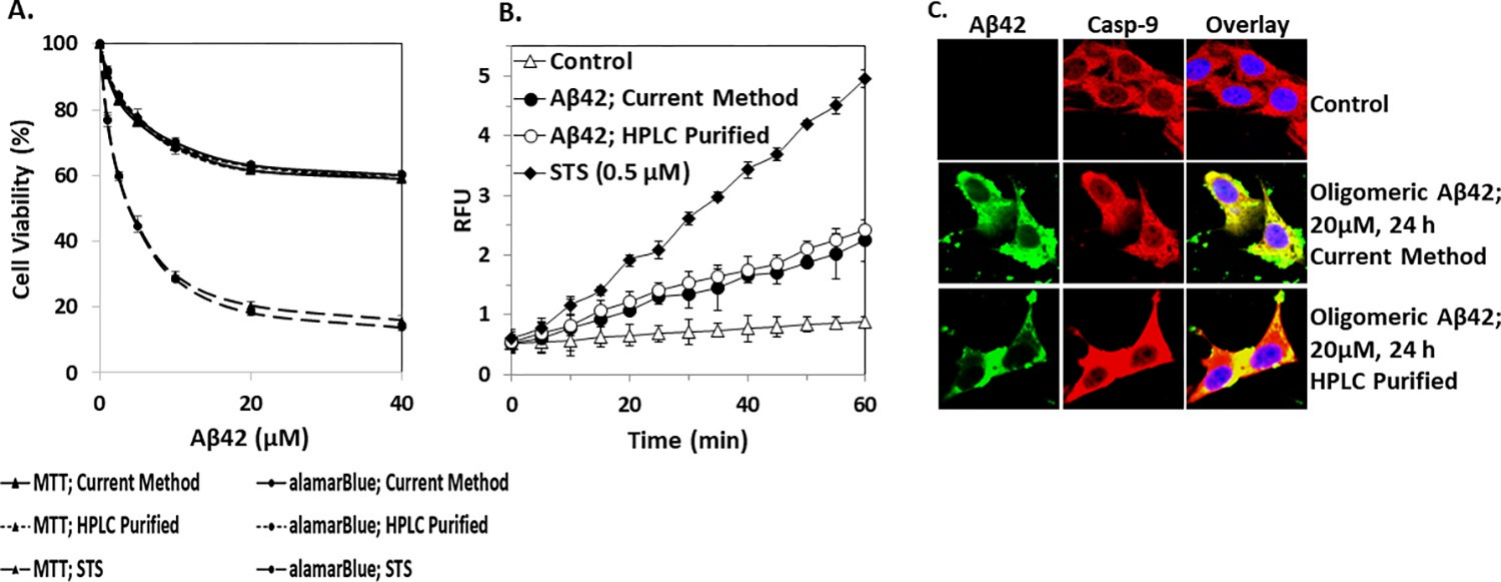
Bioactivity investigations of purified Aβ42. (A) Cytotoxicity of Aβ42 was measured in HeLa cells by MTT reduction assay and alamarBlue assay where staurosporine (STS) was used as control. (B) DEVDase activity was measured with 10μM ac-DEVD-AMC substrate in HeLa cells. Cells were treated with 20μM oligomeric Aβ42 for 2+22 h. Staurosporine (STS)-treated (0.5μM for 6 h) cells were used as a positive control. Negative controls were the cells which were not treated with the peptide or STS. RFU indicates relative fluorescence unit. (C) Confocal microscope images of Aβ and caspase-9 in HeLa cells where monoclonal mouse anti-Aβ (6E10) and polyclonal rabbit anti-caspase-9 (p10) antibodies were applied to identify Aβ (green) and caspase-9 (red) which were visualized by using secondary goat anti-mouse IgG and goat anti-rabbit IgG antibodies, respectively. Nuclei were stained with DAPI (blue). Yellow spots indicate the interaction of caspase-9 and Aβ42. Control is cells incubated without Aβ42 peptide. Only representative cell images are presented here. The results are the mean ± standard deviation of three independent experiments.

Different biological properties of purified Aβ42 peptide were checked and compared with the HPLC-purified peptide to check its competitiveness. Cytotoxicity of Aβ42 was measured by MTT and alamarBlue assay (Fig 3A). DEVDase activity was measured with 10μM Ac-DEVD-AMC substrate (Fig 3B). Interaction with caspase-9 was checked by confocal microscope images. Yellow spot indicates the interaction of caspase-9 and Aβ42 (Fig 3C). Cytotoxicity to HeLa cells, DEVDase activity in HeLa cells, and interaction with caspase-9 were found compatible with previous reports and HPLC-purified peptide [25–27].

## Discussion

As chemically synthesized peptides are expensive and complex to produce thus reduces the extent of experiments to understand the aggregation and toxic properties of Aβ42 assemblies and may have deprived the new investigators from exploring Aβ42 peptide associated pathologies, though notable development has been achieved in understanding the assembly mechanism of Aβ42 peptide and its toxic properties by using chemically synthesized peptides [28]. Thus, a highly productive, economic and simple purification method of Aβ42 peptide is very much essential for AD associated researches and that can be achieved by recombinant protein purification system.

For greater yield and purity, preparation of inclusion body of fusion protein is useful [29]. Usually, insoluble inclusion bodies are recommended to solubilize in strong denaturants like urea which hinders peptide precipitation by restricting inter and intra-molecular interactions (β-sheet content) [30]. Removal of urea is important for the maximum activity of the selected protease which may cause some difficulties in purification by facilitating agglomeration of the peptide. Previous studies reported different techniques to counterfeit this problem [31]. Among those, on-column cleavage [12, 31] is an important one which is tiring, costly and may require additional purification step. Active enzyme in presence of urea may avoid these obstacles [30, 32]. Previous research showed that Usp2-cc [33], a deubiquitylating enzyme, in presence of 3–4 M urea also it can retain its activity [15]. This enzyme is reported to cleave off the peptide from a fusion partner consisting of ubiquitin in between fusion protein and target peptide in presence of urea [15]. Initially, after digestion with Usp2-cc enzyme, RP-HPLC method was used to purify Aβ42 peptide but we faced some troubles with HPLC column regeneration which made the process difficult though the quality and yield was good. We thought by reducing the amount of fusion GroES-Ubiquitin protein in the injected sample to HPLC, we can minimize this issue. To reduce the burden on the HPLC column, later an additional nickel column affinity chromatography was involved for the initial purification where cleaved Aβ42 peptide was recovered from the “flow through” portion as histidine tagged fusion GroES-Ubiquitin protein was bound in the nickel column. Later for the removal of urea and other salts, RP-HPLC was used but our aim was not served as the previously experienced problems were still there. Then isolation of the fusion GroES-Ubiquitin protein from cleaved Aβ42 peptide based on their solubility profile was attempted.

After trying with different solvents in different combinations (Table 1), finally we found that in presence of 1.5 M urea and 50% methanol, GroES-Ubiquitin protein was precipitated where Aβ42 peptide was present in the supernatant. Upon isolating the Aβ42 peptide, the main challenge was to remove the solvent without using chromatography. We tried using a molecular weight cut-off membrane to remove both the salt and solvent, but this method was inefficient due to the large sample volume. Additionally, complete solvent removal was not possible. We also considered dialysis, but this would necessitate another chromatography step to remove the solvent which we wanted to avoid. Then we considered using a rotary evaporator, but we were concerned that the time and temperature conditions would favor Aβ42 aggregation. We hypothesized that since 1.5 M urea was present in the supernatant, its concentration would gradually increase during evaporation, effectively preventing Aβ42 aggregation. This approach worked as expected which was the ultimate solution of our objective for the removal of tedious chromatography techniques from the Aβ42 peptide purification method.

**Table 1:** Precipitation of fusion protein with different solvents at different concentrations.

Digested fusion protein with 3M urea at room temperature		Presence of Aβ42 and other proteins in different fractions		
Solvents	Final Concentration (%)	Precipitate	Supernatant	
			Aβ42	Remarks
Acetonitrile	10%	All proteins were present; no separation; excluded for further investigation	yes	Presence of other proteins
	25%		yes	Pure Aβ42; yield was too low
	50%		no	Not considered for further investigation
	75%		no	
	90%		no	
Ethanol	10%		yes	Presence of other proteins
	25%		yes	Pure Aβ42; yield was too low
	50%		no	Excluded for further investigation
	75%		no	
	90%		no	
Methanol	10%		yes	Other proteins were also present
	25%		yes	Other proteins were also present
	50%		yes	Pure Aβ42; high yield
	75%		yes	Pure Aβ42; yield was low
	90%		yes	
Isopropanol n-Butanol Acetone	10%		no	Excluded for further investigation
	25%		no	
	50%		no	
	75%		no	
	90%		no	

Final challenge was the removal of different salts present in the dried powder. We then experimented with different percentages of methanol, ethanol, isopropanol, and acetone, but these attempts were unsuccessful. The presence of even a small amount of water kept the peptide in its solubilized form due to the presence of urea in the dried powder. Then we planned to use solvents without involving water and in doing so pure methanol, ethanol, isopropanol and acetone were employed. Among them isopropanol and acetone were not efficient enough in removing salts. With methanol, both the salts and the peptide dissolved into the solubilized fraction, which was not favorable for getting a higher yield. 100% ethanol was effective in dissolving the salts but not the peptide, which provided a solution. However, after the first wash, salt removal was insufficient. We repeated the washing three times, but some residues remained. Finally, we used 100% methanol for the final wash, which removed all the residuals very effectively. This time, the Aβ42 peptide did not dissolve into the solubilized fraction, as the ethanol washing had effectively removed the urea.

Various biophysical characterizations and bioactivity assessments were conducted to demonstrate its competitiveness with other peptides. Data from the Th-T assay, oligomer formation, β-sheet formation, and fibril formation confirmed its physical characteristics, which are crucial to proving its efficiency. Moreover, various cytotoxicity assessments, caspase assays, and the interaction between caspase-9 and Aβ42 were examined and found to be comparable with previous reports. These assessments are crucial to understanding its competency in exerting the expected cellular events. Interestingly, using the peptide purified by described method, different research articles have been published from different parts of world where different underlying mechanisms of amyloidopathy caused by Aβ42 have been studied [2, 34–36].

Among the available recombinant purification methods [15, 17–19, 37–44], considering the simplicity, time of purification and final yield of purified peptide, this current method is the optimal solution from every point of consideration. All limitations like involvement of expensive equipment, process complexity, poor yield etc. encountered previously for Aβ42 peptide purification are eliminated here. All the previously described methods used different sophisticated techniques including affinity chromatography, SEC, HPLC, cutoff membrane filtration, lyophilization and so on. But the described process is so simple to execute as no such special technique is involved here. It successfully eliminates the requirement of using special equipment like HPLC, FPLC and freeze dryer which makes the process more economic and feasible. Avoidance of lyophilization process reduces the total time of purification also. Moreover, interestingly the final yield is 20 mg from 1-liter bacterial cell culture which is outstanding. Previously HPLC-purified Aβ42 peptide [15] was used to compare the purity and competitiveness in terms of physicochemical properties and bioactivity characteristics and no significant deviation was found with the Aβ42 peptide purified by the current method.

## Conclusion

The method used here is the most convenient, fast, economic and unique for the purification of Aβ42 peptide among different procedures adopted in previous. In our lab, about 200 mg Aβ42 peptide was purified just within 5 days which indicates that this method can be utilized for the large-scale production also. The final product is just ready to use as it is already monomerized by using HFIP. This innovation hopefully will cease the necessity of doing further research on Aβ42 peptide purification.

## Supporting information

S1 Raw images Original SDS-PAGE data of Fig 1B. (PDF)

S1 Fig MS data of Aβ42 peptides purified by current method and HPLC. (PDF)

S2 Fig RP-HPLC data of HPLC purified Aβ42 peptide. (PDF)
