# Supplementary figures and images for: An expeditious and facile method of amyloid beta (1–42) purification
Source: PLoS One. 2024 Jul 11;19(7):e0307213. doi: 10.1371/journal.pone.0307213 (PMC11239053; doi:10.1371/journal.pone.0307213)

**A.**

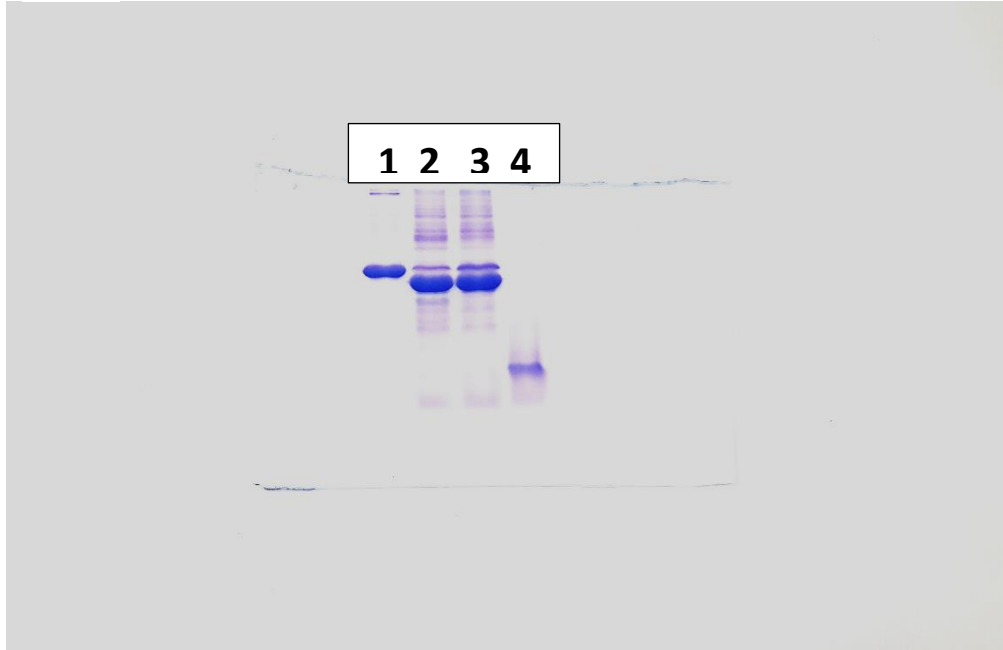

**B.**

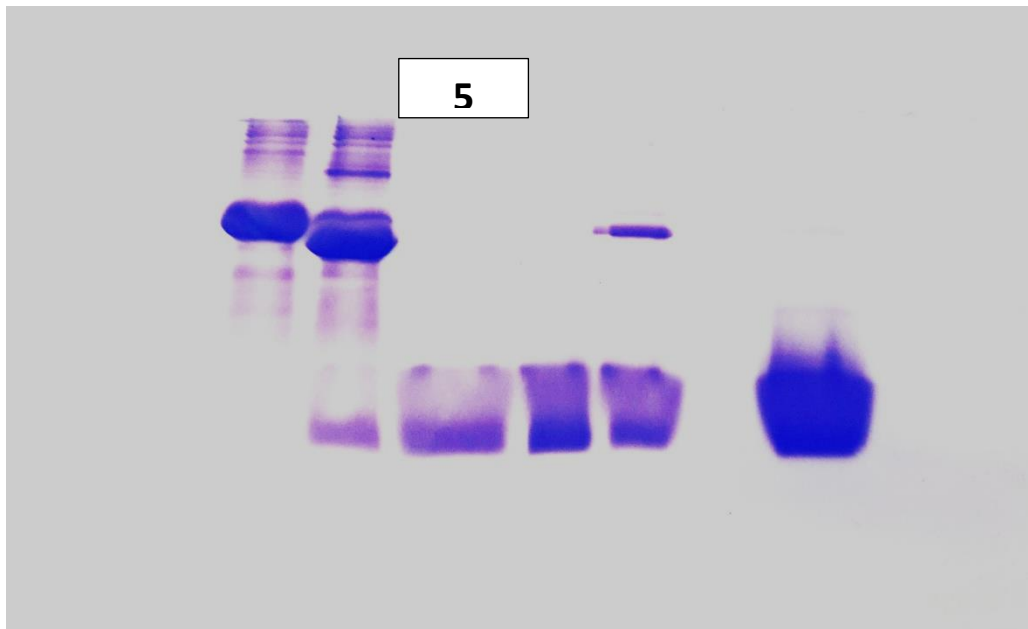

**Fig S1. Original SDS-PAGE data of Fig 1(B).** (A) Sample 1, 2, 3 & 4 of Fig 1(B). (B) Sample 5 of Fig 1(B)

Supplement: S1 Raw images — (PDF) [file pone.0307213.s001.pdf]

**A.**

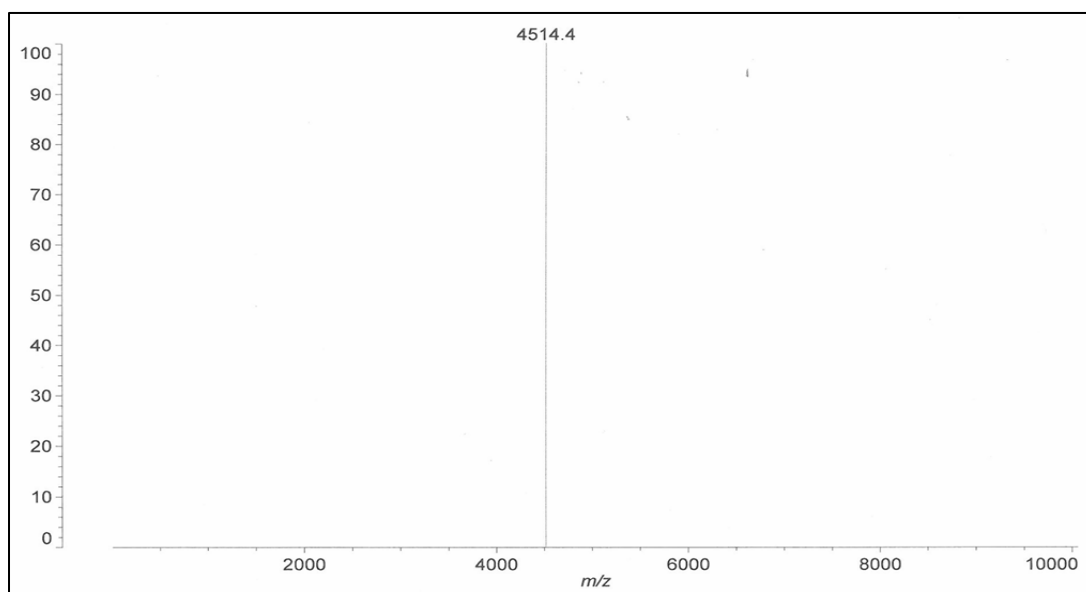

**B.**

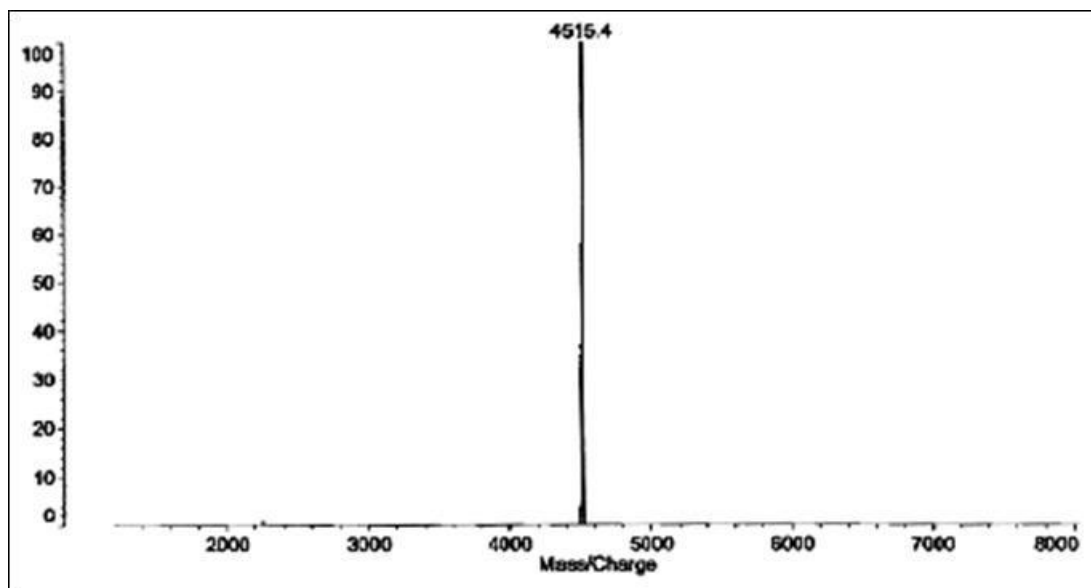

**Fig S2. MS data of A $\beta$ 42 peptides purified by current method (A) and HPLC (B).**

Supplement: S1 Fig — (PDF) [file pone.0307213.s002.pdf]

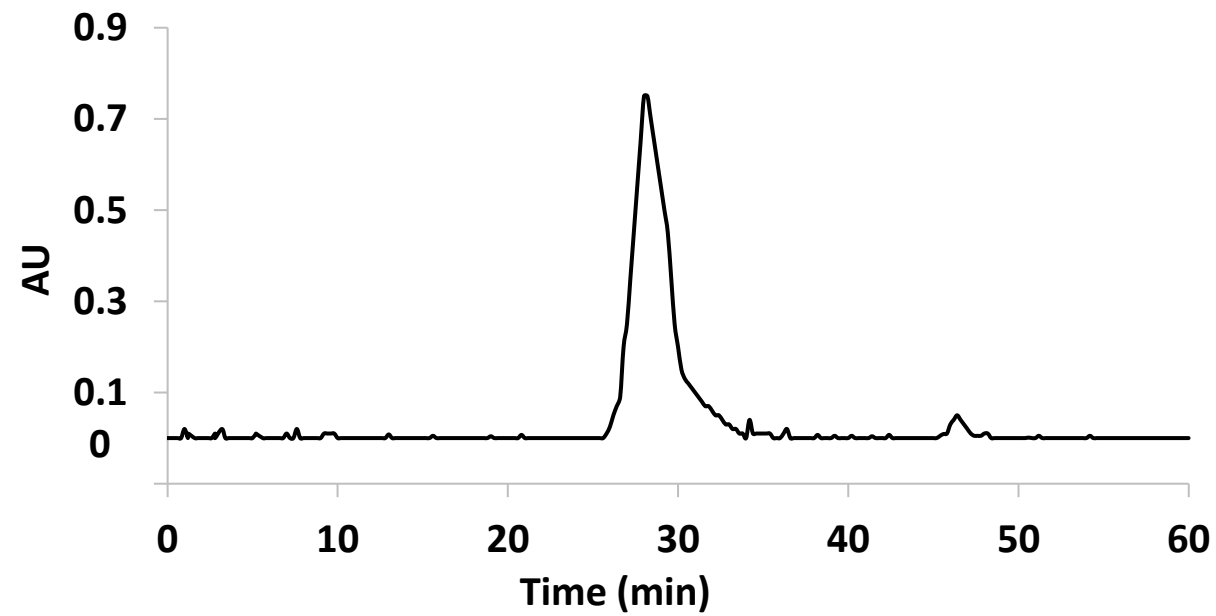

**Fig S3. RP-HPLC Data of HPLC Purified A $\beta$ 42 peptide**

Supplement: S2 Fig — (PDF) [file pone.0307213.s003.pdf]
